# Local anesthetics as a therapeutic tool for post COVID-19 patients: A case report

**DOI:** 10.1097/MD.0000000000029358

**Published:** 2022-07-15

**Authors:** David Vinyes, Montserrat Muñoz-Sellart, Teresa García Caballero

**Affiliations:** a Institute of Neural Therapy and Regulatory Medicine, Sabadell, Barcelona, Spain; b Campus Docent de Sant Joan de Déu, Barcelona, Spain; c Neural Therapy Research Foundation, Sabadell, Barcelona, Spain.

**Keywords:** case report, local anesthetics, postCOVID-19, procaine, neural therapy

## Abstract

**Background:**

Post COVID-19 syndrome (PC-19S) appears to be independent of acute illness severity and humoral response. The involvement of the autonomic nervous system (ANS), expressed by dysautonomia, has been hypothesized as a contributor. Several studies have associated the therapeutic effects of local anesthetics (LA) to their action on the ANS. To the best of our knowledge, this is the first report of therapeutic injections with LA linked to clinical improvement in a patient with PC-19S.

**Patient concerns and diagnoses:**

This was a 54-year-old-man with postCOVID-19 symptoms lasting 14 weeks, including fatigue, breathlessness, diarrhea, muscle pain, and emotional lability.

**Interventions and outcome:**

Injections of 0.5% procaine in the stellate ganglion (SG) and sphenopalatine ganglion (SPG), and in clinically relevant points in the scalp, thorax, and abdomen were performed 3 times over 3 months. The patient reported progressive improvement and was asymptomatic upon completing the treatment. Prescribed medications were discontinued. The 36-Item Short Form Health Survey (SF-36) score showed significant improvement across all SF-36-domains.

**Conclusion:**

Subanesthetic doses of LA injected in clinically relevant points led to rapid and complete symptom resolution in this patient with PC-19S. Targeted LA injections may have therapeutic benefits in PC-19S and in other chronic diseases linked to stress and inflammation.

## 1. Introduction

SARS-CoV-2 infection in its acute phase can cause a wide range of clinical syndromes, from asymptomatic infection to severe respiratory disease. It can be accompanied by general symptoms caused by direct effects of the virus or by an activation of inflammatory and clotting cascades.^[1]^ The persistent form of the disease has been given several names, including postCOVID-19 syndrome (PC-19S), postacute COVID-19 syndrome, and long-COVID-19. In October 2021, the WHO defined postCOVID-19.^[2]^ In 1 follow-up study, more than 70% of patients had at least 1 symptom 6 months after their acute infection.^[3]^ The most common symptoms are muscle fatigue or weakness (63%) and sleeping difficulties (26%, see Table 1). Even asymptomatic patients were at risk of developing symptoms several weeks later. In addition, many patients present symptoms similar to a chronic dysfunction of the autonomic nervous system (ANS). Vasomotor, sudomotor, gastrointestinal, pupillomotor disorders, etc are common in these patients. It has been postulated that therapeutic agents targeting the ANS may improve patient symptoms.^[4]^

**Table 1:** Commonly described symptomatology in postCOVID-19 patients.^[3]^

Fatigue
Headache
Cognitive impairment (“brain fog”)
Dyspnea
Orthostatic intolerance
Palpitations/tachycardia
Temperature intolerance
Labile blood pressure
New-onset hypertension
Gastrointestinal symptoms (e.g., abdominal pain, bloating, nausea)
Symptoms of mast cell activation syndrome (e.g., pruritis, urticaria, flushing, angioedema, wheezing, gastrointestinal symptoms, tachycardia, labile blood pressure)
Sleeping difficulties

Dysfunction of the ANS, with hyperactivity of the sympathetic nervous system (SNS), may heighten the acute inflammatory response. Consequently, it may be possible to regulate this hyperinflammation by modulating the SNS. Clinical and experimental data point to reductions in proinflammatory cytokines when LA injections are used to block SNS structures, as in stellate ganglion (SG) block treatment.^[5]^ There are different ways to explain the variety of therapeutic targets of these compounds, including their ability to modify the physical characteristics of the cell membrane.^[6]^ Based on the presumed antiinflammatory, neuroprotective, immunomodulatory and antiviral effects, improved perfusion and antithrombotic effects of LA agents,^[7,8]^ we performed LA injections in a patient with PC-19S, in targeted areas where SNS hyperactivity was suspected on clinical grounds.

Procaine is an ester-type LA with a half-life of ~20 minutes. It is one of the least neurotoxic LA, and while systemic toxicity is dose-dependent,^[9]^ commonly used doses of 0.5% procaine are quite low and pose minimal risk. The maximum dose that was administered to our patient was 110 mg in 1 session, which is quite far from the maximum weight-based dose (750 mg).

To the best of our knowledge, this is the first report of therapeutic injections with LA linked to clinical improvement in a patient with PC-19S.

## 2. Case history

At the first visit in July 2021, a 54-year-old Caucasian man reported fatigue, breathlessness, emotional lability, and outbreaks of diarrhea and muscle pain (VAS 10/10) for 14 weeks (Table 2). These symptoms appeared 2 weeks after the onset of an asymptomatic COVID-19 infection, as confirmed by positive PCR and a subsequent SARS-CoV-2 IgG serology (134 AU/mL). The patient was referred to a rehabilitation service and the immunology department but his clinical course did not improve. He was prescribed paracetamol and anxiolytics that he decided not to take.

**Table 2:** Timeline of care.

Date	Event
2021/3/10	COVID-19 (PCR +) asymptomatic
2021/3/25	Onset of symptoms
2021/7/13	First session with LA
2021/7/26	Follow-up visit and second session with LA
2021/9/09	Follow-up visit and third session with LA
2021/11/05	Follow-up visit

He had a history of childhood tonsillectomy and viral meningitis. He sustained fractures of the frontal bone and the clavicle, radius, ulna, scaphoid and ribs on the left side in an accident at age 21. He also sustained a traumatic brain injury requiring drainage of a left subdural hematoma at 43. Other medical history included recurrent herpes labialis, diverticulitis, chronic diarrhea and hypertension treated with an angiotensin II receptor antagonist with suboptimal control. He did not present with tachycardia or orthostatic hypotension.

Laboratory analysis in August 2021 revealed elevated ALT (113 U/L, ref. 5–40 U/L), GGT (159 U/L, ref. 5–40 U/L), alkaline phosphatase (42 U/L, ref. 46–116 U/L), aldolase (8 U/L, ref. 0.3–6.0 U/L), and serology testing was positive for parvovirus B19, herpes simplex and Epstein-Barr virus (all positive IgG). Total complement activity (CH50) was also elevated (>65 U/L, ref. 28–60 U/L), and 25-hydroxyvitamin D was deficient (23.3 ng/ml, insufficiency ref. 20–29,9 ng/ml). Antinuclear antibody (ANA) and smooth muscle antibody (SMA) tests were also positive (160 URP and 40 URP, respectively). No signs of pathology were observed on gastroscopy, colonoscopy, nor electromyography.

This patient had several of the symptoms listed in the definition of PC-19S. These symptoms appeared 1 month after infection and lasted for more than 2 months; this cannot be explained by an alternative diagnosis.

## 3. Intervention and follow-up

After obtaining informed consent form and completing the SF-36 questionnaire, the left SG was injected on his initial visit. Injections were done under the areas of tension or pain identified by palpation in the scalp (3 mL), the left frontal scar (0.5 mL), and the tonsillectomy scar. Infraorbital and mental nerves were injected using the intraoral approach. In addition, 2 mL was injected into myofascial tension points (MTP) in the trapezius muscles, 5 mL in MTPs in the chest wall and abdomen, and 3 mL was injected intraabdominally via the umbilicus (Table 3). Injections contained 0.5% procaine with hydrochloric acid as an excipient (Lab: Procaine Serra) using a 5-mL syringe with fine needles 0.4 × 25 mm and 0.4 × 40 mm (27G 1 ½ Sterican Braun).

**Table 3:** LA injections, reason and chronogram.

		Visit		
Intervention	Reason	1st	2nd	3rd
Left SG	History of traumatic injury in left SG area	X	X	
	Sympathetic neurogenic inflammation processes			
Scalp tension points	Pain in scalp, fatigue, and brain fog	X	X	
Frontal scar	History of traumatic injury	X	X	
Tonsillectomy scar	History of tonsillectomy	X		
Infraorbital and mental nerves	History of herpes labialis	X	X	
Trapezius tension points	Myalgia and fatigue	X	X	X
Thorax painful points	Thoracic somatovisceral reflex	X	X	
Abdomen painful points	Abdominal somatovisceral reflex	X	X	X
Intraabdominal	Autonomic control in abdominal organs	X	X	X
Left SPG	History of left head injury and hypertension		X	
	Parasympathetic modulation for headache and blood pressure			
Left tonsil pillar	Pain in left amygdala		X	
Left supraorbital nerve	Pain in left scalp		X	
Occipital tension points	Pain in left occipital area			X
Cervical tension points	Myalgia and fatigue			X
Bilateral L1 spinal	Autonomic control in abdominal organs		X	X
Intravenous	Antiinflammatory, simpatholytic, and vasodilating effect			X

Left SG block^[10]^ was performed using palpation to identify the anterior tubercle of the left transverse process of the sixth cervical vertebra (Chassaignac tubercle). A Braun 0.4 × 25 mm needle was directed 45 degrees caudally, medially, and dorsally and inserted to a depth of 2 cm. After negative aspiration, 2.5 mL of procaine 0.5% was slowly injected. Correct injection technique was confirmed by transient ipsilateral Horner syndrome (ptosis, myosis, enophtalmos), increased vascularity of the left sclera, and simultaneous feeling of warmth in the left half of the head and the ipsilateral upper extremity.

At the second consultation, 2 weeks later, the patient reported general improvement with a marked decrease in diarrhea episodes. When abdominal discomfort with diarrhea did appear, it was followed by general malaise, myalgias (VAS 7/10), a sensation of pain in the scalp, and pain in the left occipital area and left amygdala when swallowing. He had a herpes labialis eruption but with less intensity than usual.

Presuming that the symptoms manifested by the patient may be related to dysautonomia, during this second visit, the points of the previous sessions were injected again, and the left tonsillar pillar (1 mL), left SPG (2 mL), left supraorbital (0.5 mL), and bilateral L1 spinal ganglion (2 ml) were added (Table 3). The patient was completely pain-free immediately after the injections.

To perform the SPG block^[11]^ we used a high tuberosity approach with a Braun 0.4 × 40 mm 27-G needle attached to a syringe and bent to 60º at 3.5 cm from the tip. The patient was asked to partially open his mouth while lying down. After palpating the posterior surface of the zygomatic process of the maxilla, the needle was entirely inserted at a height of the mucobuccal fold above the maxillary second molar at a 45-degree angle directed superiorly, medially, and posteriorly toward the pterygopalatine fossa; 2 mL was delivered after aspirating.

The L1 spinal ganglion was treated while the patient was seated, leaning slightly forward, by completely submerging a 0.4 × 4 cm 27-G needle 1 finger width lateral to the L1 spinous process (vertically through the muscle mass). After aspirating, we introduced 2 mL of 0.5% procaine near the transverse process. The procaine effect reaches the spinal ganglion via the membrane line and the sympathetic trunk via the communicating rami according to Mink.^[12]^

The patient reported a clear improvement immediately after the intervention and remained symptom-free for 3 weeks, with mild myalgia (VAS 3/10), after the second session, and abdominal pain and diarrhea that reappeared after this period, again followed by myalgia (VAS 6/10) and fatigue but with much less intensity. He did not present new outbreaks of herpes labialis nor did he present episodes of shortness of breath or dyspnea on exertion. During this period, the Pfizer-BioNTech vaccine against COVID-19 was administered and was well tolerated. At the third visit, injections in bilateral cervical, occipital, and trapezius MTPs were performed and immediately improved respiratory inspiration. Intraabdominal, bilateral L1 spinal, and lumbar MTP injections were also given, and 2 mL of 1% procaine was administered intravenously (Table 3). The patient again reported an immediate decrease in pain after the injections.

In November, 2 months after the third visit, the patient reported “a much better outcome” with no abdominal pain or diarrhea afterwards or since. His blood pressure returned to normal (120/70 mm Hg) without any additional medications. At the onset of symptoms, the analysis showed a mild increase in liver function, vitamin D deficiency, and a low positivity of ANA and SMA. Liver function and vitamin D values were normal at 7 months after 3 AL treatment sessions.

**Figure 1.**
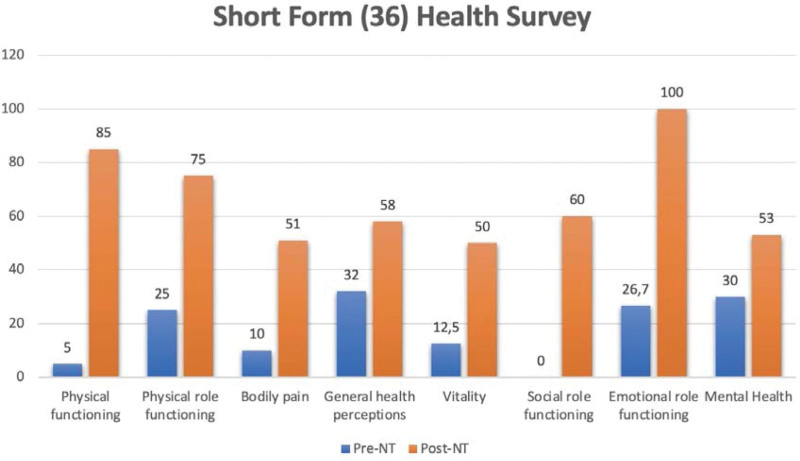
SF-36 dimensions before and after 3 sessions of treatment.

Quality of life was measured using the SF-36 questionnaire before the first visit and after the third treatment session (2 months between visits). Marked improvement across all SF-36-domains was found. The domains with the largest improvement were physical functioning (5–85), body pain (10–51), vitality (12,5–50), social role functioning (0–60), and emotional role functioning (26,7–100). Scores for physical role functioning (25–75), general health perceptions (32–58), and mental health (30–53) also showed a significant improvement (Fig. 1). No complications or side effects were detected during the treatment.

## 4. Patient point of view

“A week after asymptomatic COVID, I went to sleep 1 day as usual and slept for about 10 hours straight with no apparent explanation. From then on, severe muscular pain started all over my body, but more concentrated in my legs and arms as well as a deep and general fatigue and intense diarrhea. I had the occasional mildly good day, but in general the crises of pain, exhaustion, and diarrhea occupied a large part of the week without finding an effective treatment. I tried to do some exercise (walking with great effort and half shuffling my feet). I took 3 paracetamol tablets a day for over a month without feeling any improvement so I stopped taking it. It was 3½ months of deep fatigue and anxiety. My social and professional agenda became empty as the crises appeared without warning. It was then that I started my first neural therapy session. I was injected with a local anesthetic in various parts of my body and within a few days I felt a general improvement, the attacks became less and less intense until they completely disappeared 3 months later. I have returned to my normal life: I feel good physically and emotionally, and I am trying to get back in shape.”

## 5. Discussion

PC-19S is independent of the severity of acute illness and humoral response.^[3,13–17]^ This case report describes a patient who, after asymptomatic SARS-CoV-2 infection, subsequently developed symptoms described in patients with PC-19S. This case is interesting from the perspective of symptom control via injection of a low concentration of LA (0.5% procaine) in specific points with a therapeutic goal other than local anesthesia. Injections were done in different locations according to clinical symptoms and patient history (i.e., neural therapy).^[18]^ The development of person-centered interventions for recovery and rehabilitation is highly valued by patients suffering from PC-19S.^[19]^ This case aims to illustrate the possibility of using LA as an alternative for the control of symptoms in patients affected by postCOVID-19 together with other therapies indicated for this purpose. Although it is only a case report and it is not possible to establish any causal relationship between the intervention and the improvement, the close temporal relationship between the intervention and the patient’s improvement suggests a therapeutic effect of LA injection in this patient. Well-designed studies should be carried out to evaluate the results obtained.

### 5.1. Inflammation

Clinical and experimental data show that repeated, temporary SG blocks with procaine may regulate sympathetic-triggered neurogenic inflammation processes, via the following mechanisms: reduction of natural killer cell activity and of inflammatory cytokines (IL-1, IL-4, IL-6, IL-8, TNF-a), increase of antiinflammatory cytokines (IL-10 and of CGRP), improved endothelial function and microcirculation, and reduced coagulopathy and other pathophysiologic positive feedback loops. The technique we used for SG block is not a purely sympathetic block, since the vagal fibers are also influenced by this injection, in part because of their anatomical proximity, in part through the communicating branches. Both components, sympathetic and vagal, are essential for the transient interruption of immune and inflammatory reflex pathways.^[8]^

Inflammatory diseases can contribute to various digestive diseases. One explanation for this effect is that LA drugs act on inflammatory and immunocompetent cells and also block hyperreactive autonomic nerve pathways.^[5]^ SG blocking relieves abdominal pain and reduces inflammatory factors— 1 study reported a reduction in IL8 levels in patients with ulcerative colitis.^[20]^ The sympathetic nerves of the splanchnic routes lead to the autonomic control of the visceral organs.^[21]^ In our case, in addition to the SG, the L1 spinal ganglion area and MTP in the abdomen were injected and an intraperitoneal injection was performed, thus contributing as a whole to the control of abdominal symptoms. Reviewing the literature on the effects of SG block, we found that a reduction of neurogenic pulmonary edema and pulmonary arterial hypertension has also been observed.^[8]^ Therefore, injection of the SG may act at the functional level of the lung, thus improving respiratory symptoms.

### 5.2. Pain

The persistence or appearance of pain is a common long-term complication of COVID-19. A cohort study evaluated symptoms 2 months after the symptomatic onset of COVID-19 and found that a large proportion of individuals had joint pain (27.3%) with chest pain (21.7%) and myalgia to a lesser extent^[15]^ The mechanism of muscle involvement of SARS-CoV2 is not yet fully understood. The virus can directly cause muscle fiber atrophy and necrosis; neuronal demyelination can also contribute to muscle weakness. Cytokines and pro-inflammatory signaling molecules can contribute to the alteration of skeletal muscle tissue.^[22]^ Finally, impairment of nociceptive receptor flexibility can also generate pain, induce neuropathies, and worsen existing pain.^[23]^ LA injections can reduce the severity of pain, and this effect can persist for several weeks. Injection in multiple specific sites appears to offer a greater pain reduction than in a single site.^[24]^ An immediate response with decreased pain after injecting with LA increases the probability of pain control over the medium term. These data are similar to the evolution of our patient who reported a decrease in pain after each treatment; the subsequent periods of pain were shorter and less intense until they completely resolved. Injection of MTP with LA is associated with symptom improvement. In a study with women with chronic pelvic pain, injection with 2 mL 0.5% lidocaine at MTP led to a progressive improvement in pain relief.^[25]^ Visceral pain can be referred to somatic structures (skin, subcutaneous tissue, muscle), thus generating hyperalgesia, thickening of the subcutaneous tissue and thinning of the muscle. The administration of LA at MTPs, as well as the sympathetic block, can reduce the somatovisceral reflex that generates this somatic symptomatology.^[26]^ This mechanism underscores the importance of locating these individual stress points and injecting them with LA.

### 5.3. Fatigue

Fatigue is one of the most frequent symptoms in patients with PC-19S.^[2]^ Chronic fatigue syndrome has been described in association with dysfunction of the sympathetic and parasympathetic autonomic system in patients infected by SARS-CoV-2.^[27]^ It was recently suggested that the damage caused by SARS-CoV-2 to olfactory sensory neurons can lead to greater resistance to the outflow of cerebrospinal fluid.^[28]^ This may lead to accumulation of toxic substances in the central nervous system. Peripheral tissue signaling to the central nervous system might also play an important role in the control of fatigue. In a study with patients suffering from chronic fatigue syndrome, a reduction in clinical fatigue was demonstrated after intramuscular injections of 5 ml of 1% lidocaine.^[29]^ Fatigue can be caused by different mechanisms including those mentioned above. LA, in turn, can act on these potential mechanisms, potentially offering neuroprotection by regulation of the ANS. This may explain why the patient reported lessened fatigue.

### 5.4. Herpes labialis

The patient had 2 reactivations of herpes labialis, “cold sores”, after SARS-CoV-2 infection. Herpes labialis is the most common form of presentation of the herpes simplex virus type 1 (HSV-1). In a series of 80 patients with mild or moderate COVID-19, 35% of the patients reported one or more reactivations of HSV-1. Depression of the immune system, SARS-CoV-2 neurotropism, psychological stress, and fever may underlie the association between SARS-CoV-2 and HSV-1 reactivation.^[30]^ In a quasi-experimental study in patients with herpes labialis, procaine injection led to faster lesion healing: 1.6 days on average versus the 7.4 days previously reported. Here, 1.8 ml of 1% procaine was injected—half in the area of the affected nerve and half in the mucosa area immediately beneath the skin lesion.^[31]^ The evolution of our patient is consistent with previous reports, which suggest that procaine therapy may reduce symptomatic HSV-1 reactivations.

### 5.5. Blood pressure

SPG blocking has been described for more than 100 years for the control of headaches and pain in oncology.^[32]^ The physiological mechanism might be based on parasympathetic modulation that leads to a reversal of cerebral arterial vasodilation.^[33]^ In contrast, the blockade of the sympathetic neurons of the SPG can control blood pressure. In a clinical trial,^[34]^ a decrease in blood pressure was found 1 month after SPG block with 2% lidocaine in the group of patients with an overactivated sympathetic nervous system, with no disturbances in cardiac conduction or heart rate. After injection of the SPG, our patient became normotensive with continuous values of 120/75 blood pressure. This control had not been achieved with pharmacological treatment, and we suggest that treatment with 2 mL of 0.5% procaine injecting the SPG may have contributed to medium-term control of blood pressure.

### 5.6. Mood

The patient reported improved social function after treatment as measured by the SF-36 (0–60); emotional state (26.7–100) and mental health (30–53) also improved. There is growing evidence of an association between inflammation and mood disorders.^[35]^ Increased inflammatory cytokines can lead to negative mood, fatigue, anhedonia, psychomotor retardation, loss of appetite, and cognitive deficits. This association is observed in both senses so that an inflammatory state can produce changes in mood and vice versa. Therefore, it may be that inhibition of pro-inflammatory cytokines in patients who express mood variations in inflammatory processes will improve their emotional condition.^[36]^ For example, in a study with 30 patients, mood and life enjoyment were improved upon administration of a single maximum dose between 25 and 30 ml of 1% procaine.^[37]^ The antiinflammatory effect of LA in our patient also led to improvements in symptoms affecting the respiratory, digestive, and nervous systems.

## 6. Conclusions

Symptoms similar to a chronic dysfunction of the ANS are common in patients suffering PC-19S. Current knowledge on the mechanisms of action of LA suggests a role of these drugs in these patients. To the best of our knowledge, this is the first report of PC-19S treated with LA injections. LA used at doses below the anesthetic threshold may be a safe and useful treatment to control postCOVID-19 symptoms and improving QoL in these patients. Well-designed studies could elucidate the role of LA in this condition.

## Acknowledgment

We would like to thank Richard Nahas, MD, for his critical review of the manuscript and for his valuable suggestions in language.

## Author contributions

David Vinyes: Care and follow-up of the patient, bibliographic review, drafting of the manuscript, and critical appraisal of the final draft of the paper.

Montserrat Muñoz-Sellart: Bibliographic review, drafting of the manuscript, and critical appraisal of the final draft of the paper.

Teresa García Caballero: Bibliographic review, drafting of the manuscript, and critical appraisal of the final draft of the paper.

## References

[1] Wiersinga WJ Rhodes A Cheng AC. Pathophysiology, transmission, diagnosis, and treatment of coronavirus disease 2019 (COVID-19): a review. JAMA. 2020;324:782–93. 32648899 10.1001/jama.2020.12839

[2] World Health Organization (WHO). A Clinical Case Definition of Post COVID-19 Condition by a Delphi Consensus. Available at: https://www.who.int/publications/i/item/WHO-2019-nCoV-Post_COVID-19_condition-Clinical_case_definition-2021.1. [access date November 9, 2021].

[3] Huang C Huang L Wang Y. 6-month consequences of COVID-19 in patients discharged from hospital: a cohort study. Lancet. 2021;397:220–32. 33428867 10.1016/S0140-6736(20)32656-8 PMC7833295

[4] Buoite Stella A Furlanis G Frezza NA. Autonomic dysfunction in post-COVID patients with and without neurological symptoms: a prospective multidomain observational study. J Neurol. 2021;12:1–10. 10.1007/s00415-021-10735-y PMC8359764 34386903

[5] Fischer L Barop H Ludin SM. Regulation of acute reflectory hyperinflammation in viral and other diseases by means of stellate ganglion block. A conceptual view with a focus on Covid-19. Auton Neurosci. 2021;237:102903. 34894589 10.1016/j.autneu.2021.102903 PMC9761017

[6] Grage SL Culetto A Ulrich AS. Membrane-mediated activity of local anesthetics. Mol Pharmacol. 2021;100:502–12. 34475108 10.1124/molpharm.121.000252

[7] Hollmann MW Durieux ME. Local anesthetics and the inflammatory response: a new therapeutic indication? Anesthesiology. 2000;93:858–75. 10969322 10.1097/00000542-200009000-00038

[8] Grandhi RK Perona B. Mechanisms of action by which local anesthetics reduce cancer recurrence: a systematic review. Pain Med. 2020;21:401–14. 31282958 10.1093/pm/pnz139

[9] Becker DE Reed KL. Local anesthetics: review of pharmacological considerations. Anesth Prog. 2012;59:90–101; quiz 102. 22822998 10.2344/0003-3006-59.2.90 PMC3403589

[10] Puente de la Vega Costa K Gómez Perez MA Roqueta C. Effects on hemodynamic variables and echocardiographic parameters after a stellate ganglion block in 15 healthy volunteers. Auton Neurosci. 2016;197:46–55. 27143533 10.1016/j.autneu.2016.04.002

[11] Thangavelu K Kumar NS Kannan R. Simple and safe posterior superior alveolar nerve block. Anesth Essays Res. 2012;6:74–7. 25885507 10.4103/0259-1162.103379 PMC4173426

[12] Huneke J. Neuraltherapie, der chronische Schmerz im kleinen Becken. Ärztezeitschrift für Naturheilverfahren. 2000; 41, 9:602–7

[13] Moreno-Pérez O Merino E Leon-Ramirez JM. Post-acute COVID-19 syndrome. Incidence and risk factors: a Mediterranean cohort study. J Infect. 2021;82:378–83. 33450302 10.1016/j.jinf.2021.01.004 PMC7802523

[14] Anaya JM Rojas M Salinas ML. Post-COVID syndrome. A case series and comprehensive review. Autoimmun Rev. 2021;20:102947. 34509649 10.1016/j.autrev.2021.102947 PMC8428988

[15] Carfi A Bernabei R Landi F. Gemelli against COVID-19 post-acute care study group. Persistent symptoms in patients after acute COVID-19. JAMA. 2020;324:603–5. 32644129 10.1001/jama.2020.12603 PMC7349096

[16] Wang X Xu H Jiang H. Clinical features and outcomes of discharged coronavirus disease 2019 patients: a prospective cohort study. QJM Mon J Assoc Phys. 2020;113:657–65. 10.1093/qjmed/hcaa178 PMC7313792 32442308

[17] Townsend L Dyer AH Jones K. Persistent fatigue following SARS-CoV-2 infection is common and independent of severity of initial infection. PLoS One. 2020;15:e0240784. 33166287 10.1371/journal.pone.0240784 PMC7652254

[18] Rey Novoa M Muñoz-Sellart M Catalán Soriano M. Treatment of localized vulvar pain with neural therapy: a case series and literature review. Complement Med Res. 2021;28:571–7. 33845481 10.1159/000514945

[19] Kingstone T Taylor AK O’Donnell CA. Finding the “right” GP: a qualitative study of the experiences of people with long-COVID. BJGP Open. 2020;4:bjgpopen20X101143bjgpopen20X-101143. 10.3399/bjgpopen20X101143 PMC7880173 33051223

[20] Zhao HY Yang GT Sun NN. Efficacy and safety of stellate ganglion block in chronic ulcerative colitis. World J Gastroenterol. 2017;23:533–9. 28210090 10.3748/wjg.v23.i3.533 PMC5291859

[21] Cheng AV Tadi P. Neuroanatomy, White Rami Communicans. In: StatPearls [Internet]. Treasure Island, FL: StatPearls Publishing, 2021.

[22] Hasuo H Matsuoka H Matsuda Y. The immediate effect of trigger point injection with local anesthetic affects the subsequent course of pain in myofascial pain syndrome in patients with incurable cancer by setting expectations as a mediator. Front Psychiatry. 2021;12:592776. 34421663 10.3389/fpsyt.2021.592776 PMC8374945

[23] McFarland AJ Yousuf MS Shiers S. Neurobiology of SARS-CoV-2 interactions with the peripheral nervous system: implications for COVID-19 and pain. Pain Rep. 2021;6:e885. 33458558 10.1097/PR9.0000000000000885 PMC7803673

[24] Ahmed S Subramaniam S Sidhu K. Effect of local anesthetic versus botulinum toxin-a injections for myofascial pain disorders: a systematic review and meta-analysis. Clin J Pain. 2019;35:353–67. 30589660 10.1097/AJP.0000000000000681

[25] Montenegro ML Braz CA Rosa-e-Silva JC. Anaesthetic injection versus ischemic compression for the pain relief of abdominal wall trigger points in women with chronic pelvic pain. BMC Anesthesiol. 2015;15:175. 26628263 10.1186/s12871-015-0155-0 PMC4667406

[26] Jarrell J Giamberardino MA Robert M. Bedside testing for chronic pelvic pain: discriminating visceral from somatic pain. Pain Res Treat. 2011;2011:692102. 22135736 10.1155/2011/692102 PMC3216293

[27] Barizien N Le Guen M Russel S. Clinical characterization of dysautonomia in long COVID-19 patients. Sci Rep. 2021;11:14042. 34234251 10.1038/s41598-021-93546-5 PMC8263555

[28] Wostyn P. COVID-19 and chronic fatigue syndrome: Is the worst yet to come? Med Hypotheses. 2021;146:110469. 33401106 10.1016/j.mehy.2020.110469 PMC7836544

[29] Staud R Kizer T Robinson ME. Muscle injections with lidocaine improve resting fatigue and pain in patients with chronic fatigue syndrome. J Pain Res. 2017;10:1477–86. 28721090 10.2147/JPR.S139466 PMC5499959

[30] Shanshal M Ahmed HS. COVID-19 and herpes simplex virus infection: a cross-sectional study. Cureus. 2021;13:e18022. 34667693 10.7759/cureus.18022 PMC8520410

[31] Muñoz C Palacio C Posada L. Tratamiento de la infección por herpes simple: efecto de la procaína infiltrada sobre las lesiones recurrentes del herpes labial. CES Odontol. 2000; 13:20–4.

[32] Iwanaga J Wilson C Simonds E. Clinical anatomy of blockade of the pterygopalatine ganglion: literature review and pictorial tour using cadaveric images. Kurume Med J. 2018;65:1–5. 30158355 10.2739/kurumemedj.MS651001

[33] Nair AS Rayani BK. Sphenopalatine ganglion block for relieving postdural puncture headache: technique and mechanism of action of block with a narrative review of efficacy. Korean J Pain. 2017;30:93–7. 28416992 10.3344/kjp.2017.30.2.93 PMC5392662

[34] Triantafyllidi H Arvaniti C Schoinas A. Bilateral sphenopalatine ganglion block reduces blood pressure in never treated patients with essential hypertension. A randomized controlled single-blinded study. Int J Cardiol. 2018;250:233–9. 29074041 10.1016/j.ijcard.2017.10.042

[35] De Sousa Moreira JL Barbosa SMB Vieira JG. The psychiatric and neuropsychiatric repercussions associated with severe infections of COVID-19 and other coronaviruses. Prog Neuropsychopharmacol Biol Psychiatry. 2021;106:110159. 33147504 10.1016/j.pnpbp.2020.110159 PMC7605739

[36] Miller AH Maletic V Raison CL. Inflammation and its discontents: the role of cytokines in the pathophysiology of major depression. Biol Psychiatry. 2009;65:732–41. 19150053 10.1016/j.biopsych.2008.11.029 PMC2680424

[37] Haller H Saha FJ Ebner B. Emotional release and physical symptom improvement: a qualitative analysis of self-reported outcomes and mechanisms in patients treated with neural therapy. BMC Complement Altern Med. 2018;18:311. 30482194 10.1186/s12906-018-2369-4 PMC6258402

